# Depletion of SASH1, an astrocyte differentiation‐related gene, contributes to functional recovery in spinal cord injury

**DOI:** 10.1111/cns.13998

**Published:** 2022-10-26

**Authors:** Siyi Liu, Ge Lin, Qiao Yang, Penghui Wang, Chao Ma, Xiaowei Qian, Xiaomei He, Zhangji Dong, Yan Liu, Mei Liu, Ronghua Wu, Liu Yang

**Affiliations:** ^1^ Department of Neurosurgery Affiliated Hospital of Nantong University Nantong China; ^2^ Key Laboratory of Neuroregeneration of Jiangsu and Ministry of Education, Co‐innovation Center of Neuroregeneration, NMPA Key Laboratory for Research and Evaluation of Tissue Engineering Technology Products Nantong University Nantong China

**Keywords:** astrocytes, BDNF, GFAP, SASH1, spinal cord injury

## Abstract

**Aims:**

This study aimed to evaluate the effects of the depletion of SAM and SH3 domain‐containing protein 1 (SASH1) on functional recovery after spinal cord injury (SCI) and to investigate the possible mechanism of SASH1 knockdown in astrocytes facilitating axonal growth.

**Methods:**

SCI model was established in adult rats. SASH1 small interfering RNA (siSASH1) was used to investigate its function. Hindlimb motor function was evaluated by the Basso‐Bresnahan‐Beattie (BBB) assay. The gene expressions were evaluated by the methods of qRT‐PCR, Western‐blotting, ELISA, and immunohistochemistry.

**Results:**

SASH1 knockdown improved the BBB scores after SCI and significantly reduced GFAP expression. In cultured spinal astrocytes, siSASH1 treatment decreased interferon‐γ release and increased brain‐derived neurotrophic factor (BDNF) release. When cocultured with SASH1‐knockdown astrocytes, axonal growth increased. The neuronal tropomyosin receptor kinase B (BDNF receptor) expression increased, especially in the axonal tips. SASH1 expression increased while NSCs differentiated into glial cells, instead of neurons. After SASH1 depletion, differentiated NSCs maintained a higher level of Nestin protein and an increase in BDNF release.

**Conclusions:**

These results indicate that SASH1 acts as an astrocytic differentiation‐maintaining protein, and SASH1 downregulation limits glial activation and contributes toward functional recovery after SCI.

## Graphical abstract

SASH1 expression increased significantly during NSCs differentiation into glial cells and spinal cord injury (SCI). SASH1 knockdown in primary astrocytes increased the release of BDNF and promoted axonal growth; SASH1 knockdown significantly reduced astrocytes activation and improved the recovery of function after SCI.

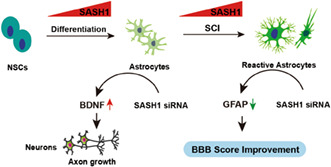

## 1 INTRODUCTION

Spinal cord injury (SCI), a devastating central nervous system (CNS) disease usually caused by traffic accidents, falls, and trauma to the spine, often results in a loss of motor and sensory function below the injury site, or even permanent paralysis.^1^ The main pathological changes in SCI are inflammation leading to demyelination, axonal degeneration, neuronal apoptosis, and necrosis. Clinical treatment focuses on the recovery of motor function and prevention of complications. To date, many studies have been conducted on the cellular and molecular mechanisms of SCI, and many effective treatment methods have been proposed. However, clinical applications are still limited owing to the complex pathogenesis and poor prognosis after SCI.^2^

Astrocytes, one of the most abundant types of glial cells in the CNS, have various functions, including neurogenesis, neuronal survival, neurotransmission, and immune surveillance.^3^ In addition to maintaining normal functions, when the CNS encounters pathological changes, such as trauma or inflammation, activated astrocytes produce a large amount of extracellular matrix, namely gliosis.^4^ Although this process protects the surrounding healthy tissue from the spread of damage, the glial scar created by gliosis eventually inhibits axon regeneration and plasticity.^5^ Therefore, researchers have used various methods to inhibit the activation of astrocytes, but they have not achieved the expected effects of promoting nerve regeneration and have even aggravated the extent of injury.^6^, ^7^ These studies indicated that astrocyte activation is an extremely complex process that needs to be clarified in SCI.

To date, lots of studies have shown that reactive astrocytes are heterogeneous. They could be described generally as helpful or harmful reactive astrocytes according to their function on neurons: the harmful astrocytes are neurotoxic since they secrete pro‐inflammatory factors leading to the rapid death of neurons and oligodendrocytes, whereas helpful astrocytes secrete neurotrophic factors that promote the survival of neurons and tissue repair.^8^ During injury‐related stress, astrocytes mediate the inflammatory response to activate the nuclear factor‐κB pathway and generate increased expression of interleukin (IL)‐1β, IL‐6, interferon (IFN)‐γ, and other neurotoxic molecules that are harmful to neuronal survival and regeneration. In contrast, in certain situations, astrocytes can increase expression of S100 calcium binding protein A10, brain‐derived neurotrophic factor (BDNF), basic fibroblast growth factor (bFGF), and other neurotrophic factors that have neuroprotective effects to promote neuron survival and tissue repair.^9^ However, SCI is a complex event involving various types of cells, and these cell types may have different outcomes and produce different pro‐ or antiinflammatory factors. Thus, activated astrocytes have dissimilar functions through various inflammatory pathways.^10^

SAM and SH3 domain‐containing protein 1 (SASH1) is a scaffold protein that belongs to the SH3‐domain containing proteins expressed in the lymphocyte family.^11^ Many recent studies have shown that SASH1 is ubiquitously expressed in most normal tissues but is decreased or absent in tumor tissues, supporting its function as a tumor inhibitory gene.^12^ Our previous study on primary cultured cortical astrocytes revealed that SASH1 small interfering RNA (siRNA) treatment remarkably decreased cell adhesion, changed cell behaviors, and was involved in the inflammatory factor high mobility group box protein 1 (HMGB1) function, suggesting that SASH1 plays an important role in astrocyte activity.^13^ Therefore, in this study, we first depleted the expression of SASH1 in rat SCI to determine whether depletion of SASH1 contributes to the functional recovery after spinal cord injury (SCI) and to investigate the possible mechanism of SASH1 knockdown in astrocytes facilitating axonal growth.

## 2 MATERIALS AND METHODS

### 2.1 Animals and SCI procedure

**FIGURE 1:**
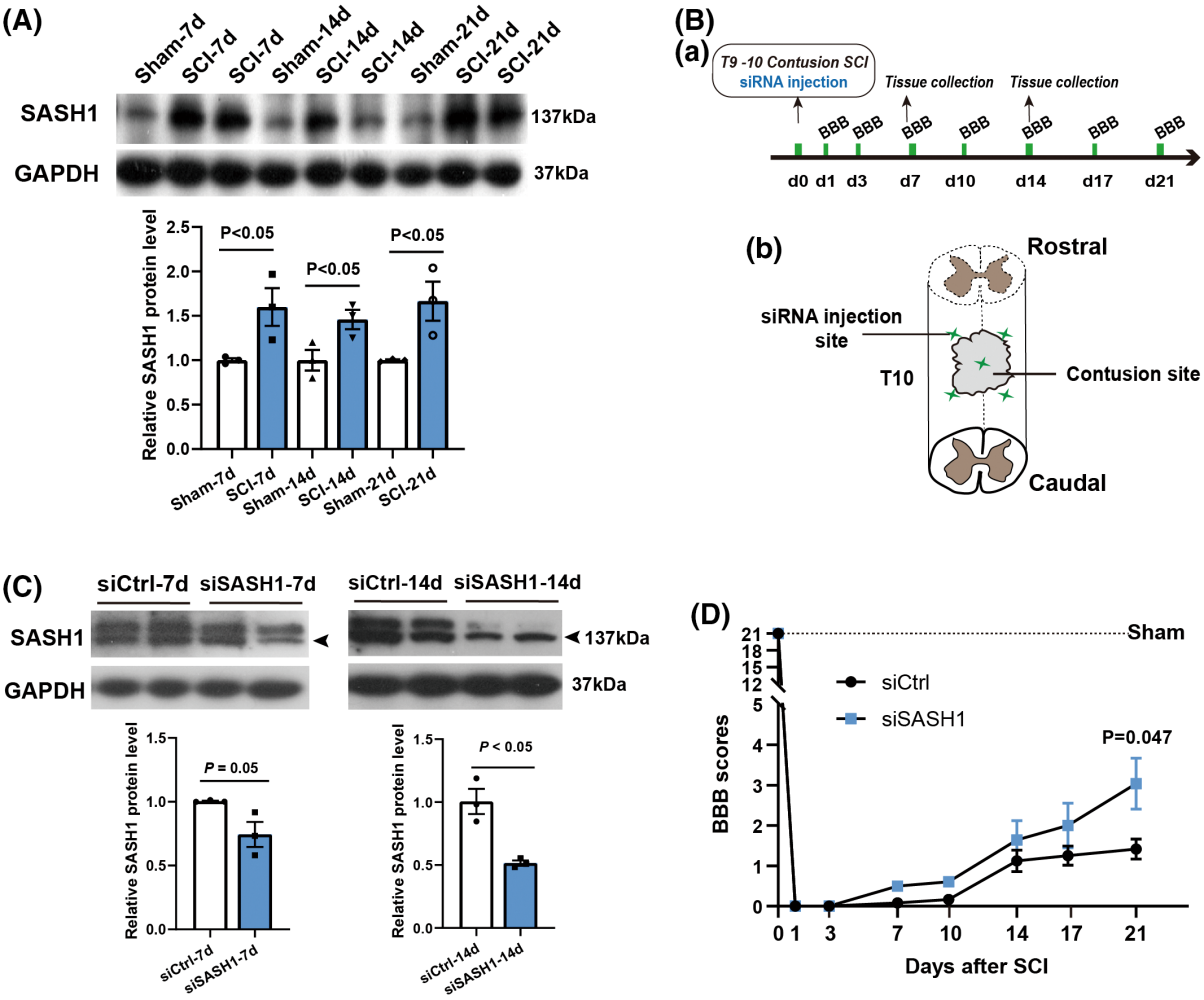
SASH1 knockdown improves rat movement recovery after spinal cord contusion. (A) Western blotting results show that SASH1 protein notably increased after spinal cord injury at 7, 14, and 21 dpi, while GAPDH was used as a loading control. Sham group: *n* = 3 rats, SCI group: *n* = 6 rats; the exact *P*‐value is presented in the figure panel, Student's unpaired *t*‐test vs. sham group. (B) Time schedule (panel a) and schematic diagram of spinal cord injury sites and siSASH1 injection sites (panel b). (C) Efficiency of siSASH1 treatment at 7 dpi (a) and 14 dpi (b). The upper panel shows the representative Western blot results. The bottom panel shows the statistical results. The relative SASH1 proteins in siCtrl‐injected rat spinal cords were normalized to 1, and those of siSASH1 injected rats at 7 dpi = 0.74 ± 0.09 and at 14 dpi = 0.51 ± 0.02 (*n* = 3). The *P*‐values are presented in the figure panel, based on Student's unpaired *t*‐tests of siSASH1 vs. siCtrl. (D) BBB scores evaluated and analyzed at 1, 3, 7, 10, 14, 17, and 21 dpi. siCtrl rats, *n* = 6; siSASH1 rats, *n* = 7, Student's unpaired *t*‐tests for siSASH1vs. siCtrl. SASH1, SAM and SH3 domain‐containing protein 1; dpi, days post‐injury; GAPDH, glyceraldehyde‐3‐phosphate dehydrogenase; si, small interfering; Ctrl, control; BBB, Basso‐Bresnahan‐Beattie; SCI, spinal cord injury.

Adult Sprague–Dawley (SD) rats weighing 200 ± 20 g were obtained from the Animal Center of Nantong University. Rats were randomly divided into three groups corresponding to 7, 14, and 21 days post‐injury, with three rats in each group. In the same manner, the sham operation groups were established for the same time points. Spinal cord contusion injury was induced as described previously with some modifications,^14^ after which the spinal cord was exposed via T9–T10 laminectomy and the vertebrae were stabilized with clamps. A moderate contusion injury was produced using an Infinite Horizon spinal cord impactor (Precision Systems and Instrumentation) with a force of 160 kilodyne (dwell time: 30 s). Postoperatively, the muscle layers were sutured, skin was secured with wound clips, and 100 μl of gentamicin was applied at the injury site to prevent infection. To investigate the role of SASH1 in SCI, dimethoxy‐modified SASH1 siRNA was synthesized and administered in vivo. For control (Ctrl) and SASH1 siRNA treatments, each rat was injected with 1 μg of siRNA at five sites. The injection locations are shown in Figure 1Bb. All exprimental procedures were performed in accordance with the guidelines of Nantong University Institutional Animal Care and Use Committee.

### 2.2 Assessment of recovery from spinal cord contusion

Behavioral Basso‐Bresnahan‐Beattie (BBB) locomotor function was assessed based on the BBB locomotor rating scale^15^ in an open field on day 0 before surgery and on days 1, 3, 7, 10, 14, 17, and 21 after surgery, where a score of 0 indicated complete paralysis and a score of 21 indicated complete mobility. Blind scoring ensured that the observers were not aware of the treatment received by individual rats.

### 2.3 RNA isolation and quantitative real‐time polymerase chain reaction

Total RNA was extracted from tissues or cells using TRIzol (Thermo Fisher Scientific, Inc.), and the RNA concentration was measured using a NanoDrop 2000 (Thermo Fisher Scientific, Inc.). Primers were synthesized by Thermo Fisher Scientific Inc. and are listed in Table 1. First‐strand complementary DNA (cDNA) was synthesized using random hexamer primers and HiScript Reverse Transcriptase (Vazyme Biotech) following the manufacturer's instructions. Real‐time qualitative (qRT) polymerase chain reaction (PCR) was performed in a total volume of 20 μl, including 1 μl of cDNA, 10 μM of primers, and 10 μl of 2 × SYBR Green qualitative PCR Master Mix (Vazyme Biotech). Glyceraldehyde‐3‐phosphate dehydrogenase (*GAPDH*) was used as an internal Ctrl.

**TABLE 1:** The sequences of primers and siRNAs

Name	Sense (5′‐3′)	Antisense (5′‐3′)
*Gapdh*	ccatcactgccactcagaagact	acattgggggtaggaacacg
*SASH1*	ggtggaactgttgcaggaat	gttggactccgtggatgact
*GFAP*	acgaacgagtccttggagag	cgatgtccagggctagctta
*PAX6*	agttcttcgcaacctggcta	ttggtgttttctccctgtcc
Control (Ctrl) siRNA	uucuccgaacgugucacgutt	acgugacacguucggagaatt
*SASH1* siRNA‐1	ccagcaguacgcagauuautt	auaaucugcguacugcuggtt
*SASH1* siRNA‐2	ggauccagcuaaugaugautt	aucaucauuagcuggaucctt

### 2.4 Western blot analysis

Cells or tissues were lysed to extract total proteins, and protein concentrations in the total cell extracts were measured using the bicinchoninic acid protein assay. Total protein samples (20 μg) were separated by sodium dodecyl sulfate–polyacrylamide gel electrophoresis, and membranes were incubated overnight at 4°C with the following primary antibodies: anti‐SASH1 (Cat: #bs‐6099R, Bioss), anti‐GAPDH (Cat: #60004‐1‐Ig, Proteintech). After washing with Tris‐buffered saline and 0.1% Tween 20, membranes were incubated with horseradish peroxidase‐conjugated secondary anti‐rabbit or anti‐mouse antibodies at room temperature for 2 h. For visualization, the immunoreactive bands were treated with a chemiluminescence solution (Cat: #180–5001, Tanon) and detected using X‐ray films. The optical density values of the target protein bands were quantified using Multi Gauge software (FujiFilm Corp. Life Science Division) and normalized to the GAPDH loading Ctrl.

### 2.5 Primary cell culture and siRNA treatment

Primary spinal neurons were isolated from the spinal cords of SD rat embryos at gestation day 14 (E14) as previously described.^16^, ^17^ Primary astrocytes of postnatal day 1 rat spinal cord were prepared as we previously described,^18^ cultured in Dulbecco's Modified Eagle Medium/Nutrient Mixture F‐12 (DMEM/F12, Cat: #11320033, Invitrogen), supplemented with 10% fetal bovine serum (Cat: #10099141C, Gibco), 0.5‐mM glutamine (Cat: #25030164, Gibco), and 1% penicillin–streptomycin (Cat: #15140122, Gibco). Cells were incubated at 37°C in a humidified atmosphere (5% CO_2_ and 95% air). To purify the cultures, when cells became confluent, they were shaken at 150 rpm for 14–16 h to remove microglial cells. E14 spinal neurons or purified astrocytes at passage 2 were used for siRNA transfection at a final concentration of 200 nM using a NEPA21 electrical transfection instrument.^19^ siRNAs were synthesized by GENERAL BIOL (Anhui, China). The sequences are listed in Table 1.

### 2.6 Immunofluorescence assay

Immunofluorescence (IF) assays were performed as described previously.^16^, ^17^, ^20^ Cultured neurons were fixed with 4% paraformaldehyde for 30 minutes. Then the cultures were washed with phosphate‐buffered saline (PBS) three times and blocked for 60 minutes. Primary antibodies against anti‐Tuj1, (Cat: #801201, BioLegend), antiphosphorylated (p)‐tropomyosin‐related kinase B (TrkB) (Cat: #ABN1381, MilliporeSigma) were incubated overnight at 4°C. After rewarming for 30 min the next day, the cultures were rinsed with PBS and incubated with the corresponding species‐specific fluorescence‐conjugated secondary antibodies at room temperature for 2 h. The cells were counterstained with Hoechst (Cat: #B2261, Sigma). Next, the cells were washed with PBS and mounted in an antifade mounting medium. Fluorescence images were acquired using a fluorescence microscope (Zeiss). For axonal length analysis, images were acquired at 20× magnification and analyzed using Image‐Pro Plus (IPP) software (version 6.0; Media Cybernetics, Inc.). We defined an axon as a cell with a neurite length > 20 μm, according to our recent publications.^16^, ^17^

For immunohistochemistry, rats were perfused with 4% paraformaldehyde, and perilesional spinal cord tissues measuring 1.5 cm were collected and cryoprotected in 30% sucrose. Then, tissue sections (16 μm) were prepared by cryostat sectioning and treated with a blocking buffer (0.4% BSA, 5% goat serum, and 0.2% Triton‐X 100 in PBS) for 60 minutes at room temperature. Primary antibodies against glial fibrillary acidic protein (GFAP) (Cat: #12389, CST), neurofilament‐200 (Cat: #N4142, Sigma), Tuj1 (Cat: #801201, BioLegend), and chondroitin sulfate proteoglycan (CSPG) (Cat: #C8035, Sigma) were incubated overnight at 4°C in a blocking buffer. After rinsing with PBS, the corresponding secondary antibodies were incubated for 120 minutes at room temperature. All the tissues were counterstained with 4′,6‐diamidino‐2‐phenylindole (DAPI) (Cat: #B9542, Sigma). The tissues were washed and mounted. Fluorescence images were obtained using a fluorescence microscope (Zeiss).

### 2.7 Enzyme‐linked immunosorbent assay

Control or SASH1‐knockdown cells were seeded on 60‐mm dishes at a density of 8 × 10^5^ cells/dish. After incubation for 24 h, supernatants from the cell cultures were harvested and analyzed using the rat BDNF enzyme‐linked immunosorbent assay (ELISA) kit (Cat: #HB500‐Ra, Hengyuan Biotechnology Co., Ltd.), rat bFGF ELISA kit (Cat: #HB1259‐Ra, Hengyuan Biotechnology Co., Ltd.), rat vascular endothelial growth factor (VEGF) ELISA kit (Cat: #HB1192‐Ra, Hengyuan Biotechnology Co., Ltd.), rat IFN‐γ ELISA kit (Cat: #HB986‐Ra, Hengyuan Biotechnology Co., Ltd.), rat LCN2 ELISA kit (Cat: #HB1260‐Ra, Hengyuan Biotechnology Co., Ltd.), and rat C1q ELISA kit (Cat: #HB920‐Ra, Hengyuan Biotechnology Co., Ltd.) following the manufacturer's instructions. The levels of multiple cytokines were measured simultaneously in each sample. The levels of the secreted cytokines were normalized to 1 in the small interfering (si)‐Ctrl group.

### 2.8 Coculture of astrocytes and neurons

For the experiments, cocultured primary neurons were plated in 24‐well plates (4 × 10^4^ cells/well), while primary astrocytes after siRNA treatment were plated in appropriate transwells (4.5 × 10^4^ cells/well) and grown until confluence. In this model, although neurons and astrocytes faced each other, they were separable, and the effect of soluble factors released from astrocytes on neurons could be studied, allowing for separate analyses of neuronal and glial populations.

### 2.9 Neural stem cell culture and differentiation into astrocytes and neurons

Neural stem cells (NSCs) were prepared from the cerebral cortex of E14 Sprague Dawley (SD) rats as described previously.^21^ The cells were maintained in DMEM/F12 medium supplemented with 2% NeuroCult SM1 Neuronal Supplement, 1% N2 Supplement‐B (Cat: #07156, StemCell), 1% penicillin–streptomycin, 2 mM glutamine, 20 ng/ml recombinant bFGF (Cat: #450–33, Proteintech), and 20 ng/ml epidermal growth factor (EGF) (Cat: #E9644, Sigma). To induce astrocytic differentiation, NSCs were seeded onto dishes in the astrocytic medium and coated with 0.1 mg/ml poly‐L‐lysine (Cat: #P4832, Sigma‐Aldrich). To induce neuronal differentiation, NSCs were seeded onto dishes in a neuron medium coated with 30 μg/ml fibronectin (Cat: #33016–015, Gibco).

### 2.10 Statistical analysis

All data were expressed as mean ± standard error. Statistical analysis was performed using GraphPad Prism software (version 8.0; GraphPad Software). Statistical significance was assessed using one‐way analysis of variance, and Student's *t*‐test was used to determine the significance of the differences between the groups. Prior to statistical analyses, the data sets for each group were tested for normality of distribution using the Kolmogorov–Smirnov test. Statistical significance was set at *p* ≤ 0.05.

## 3 RESULTS

### 3.1 Knockdown of SASH1 improved rat behavior in SCI

First, we investigated changes in SASH1 protein levels during SCI. Adult rats were randomly divided into a spinal cord contusion injury group and sham‐operated group (Ctrl). Proteins were obtained from injured rat spinal cords (total, 1 centimeter surrounding the injured center) at 7, 14, and 21 days after SCI. Western blotting was used to evaluate SASH1 protein levels, and the results showed that SASH1 protein significantly increased in SCI rats compared with Ctrl rats at the same time points (Figure 1A).

Accordingly, we then used siRNA to reduce the expression of SASH1 protein to determine its effects on SCI rat behavioral recovery. The time schedule and schematic diagram of the SCI sites and SASH1 siRNA (siSASH1) injection sites are shown in Figure 1Ba–b. For the in vivo siRNA treatment, we injected methoxy‐modified siSASH1 to allow siRNA knockdown for a longer duration in accordance with our previous study.^20^ We detected the efficiency of siSASH1, and the results showed (Figure 1Ca–b) that SASH1 protein decreased to 74% and 51% at 7 and 14 days post‐injury, respectively, compared with the control siRNA (siCtrl) group. Considering that siRNA efficiency tends to fade after 2 weeks, we collected the data until 3 weeks after SCI. The BBB test was used to evaluate hindlimb motor function. As shown in Figure 1D, BBB scores indicated better movement‐based functional recovery in siSASH1‐injected rats at 1, 3, 7, 10, 14, 17, and 21 days after SCI. To achieve consistency in the extent of injury, we performed additional dwell time for 30 seconds after spinal cord contusion. This performance resulted in a relatively lower BBB score. Even so, at 21 dpi, the siSASH1‐treated rats received a score of 3.0, which means that two joints had motion being observed. Meanwhile, the control rats received a score of only 1.4, which means that one joint was occasionally observed moving slightly. This result revealed that SASH1 knockdown could improve the hindlimb motor function recovery after spinal cord injury.

### 3.2 Depletion of SASH1 decreased GFAP expression after spinal cord contusion

**FIGURE 2:**
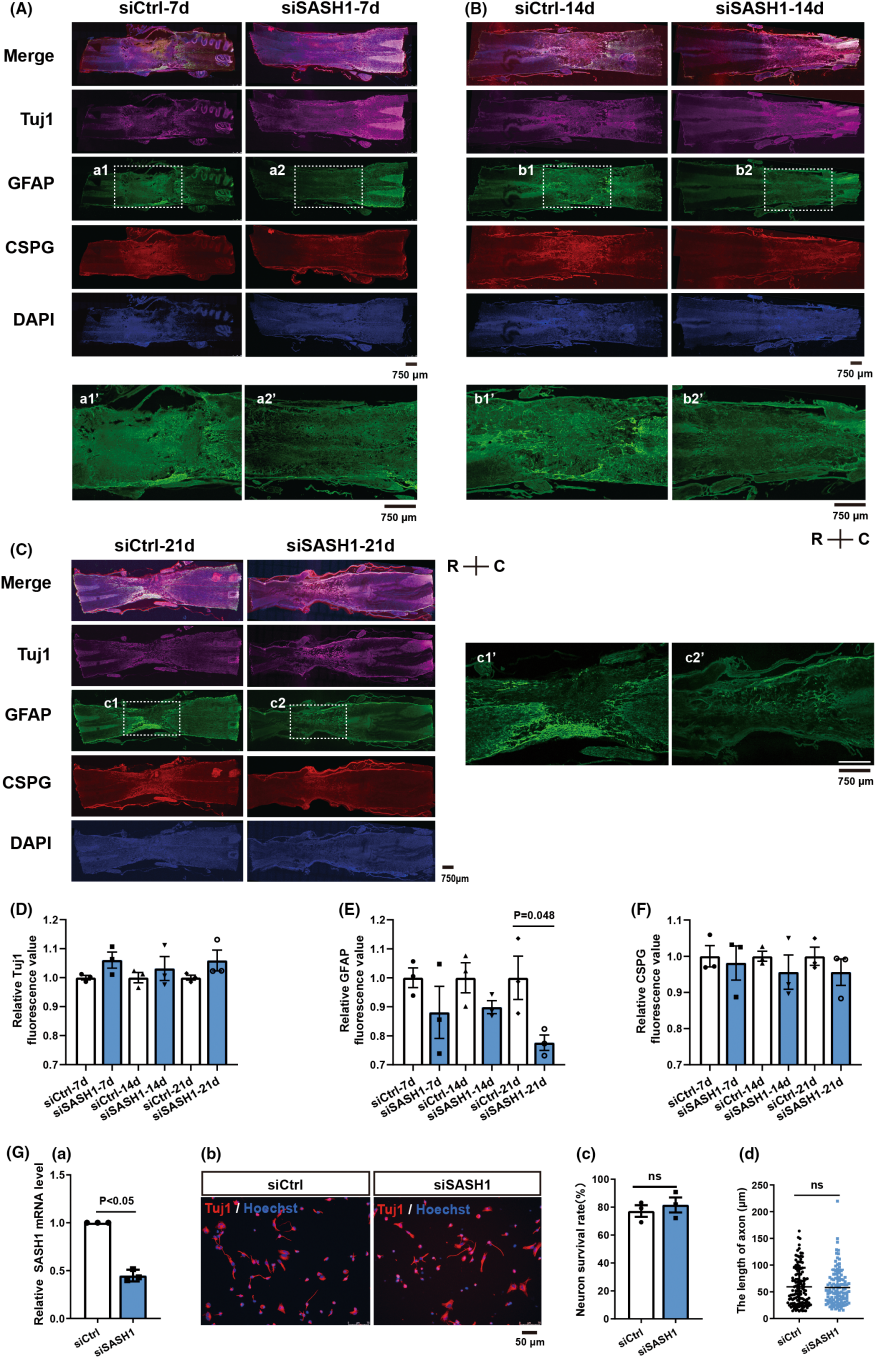
SASH1 knockdown decreases GFAP in spinal cord contusion. (A–C) Immunostaining results for Tuj1, GFAP, CSPG, and DAPI after SASH1 knockdown for 7 days (A), 14 days (B), and 21 days (C) in rat spinal cord injury. Panels a1'–a2', b1'–b2', and c1'–c2' are magnifications of the white squares in panels A a1–a2, B b1–b2, and C c1–c2, respectively. *R* indicates rostral, *C* indicates caudal, scale bar = 750 μm. (D–F) Statistical results of the average fluorescence intensity of Tuj1 (D), GFAP (E), and CSPG (F) after SASH1 knockdown at 7, 14, and 21 dpi. The fluorescence intensity in the siCtrl group was normalized to 1, and the *P*‐values are presented in the figure panel, for Student's unpaired *t*‐test of siSASH1 vs. siCtrl. (G) Effect of SASH1 knockdown in spinal neurons. Panel a, qRT‐PCR results of SASH1 expression in spinal neurons after siSASH1 treatment for 48 h (*n* = 3). Panel b, Tuj1 immunostaining results of siSASH1‐treated neurons. Panel c, Statistical result of the neuronal survival ratio (neurons stained with trypan blue dye). Panel d, statistical result of axonal length. The *P*‐value is presented in the figure panel, for Student's unpaired *t*‐test of siSASH1 vs. siCtrl. dpi, days post‐injury; SASH1, SAM and SH3 domain‐containing protein 1; GFAP, glial fibrillary acidic protein; GAPDH, glyceraldehyde‐3‐phosphate dehydrogenase; CSPG, chondroitin sulfate proteoglycan; DAPI, 4′,6‐diamidino‐2‐phenylindole; si, small interfering; Ctrl, control; d, days.

Improvement in the BBB test results suggested that SASH1 knockdown facilitated regeneration after SCI. To further investigate the effects of SASH1 downregulation, we immunostained Tuj1, GFAP, CSPG, and DAPI in the rat spinal cord after treatment with siCtrl and siSASH1 for 7 days (Figure 2A), 14 days (Figure 2B), and 21 days (Figure 2C). The average fluorescence intensity of Tuj1 in the injured area of the siSASH1 group was increased (Figure 2D) and that of CSPG was reduced (Figure 2F) compared with that of the siCtrl group at 7, 14, and 21 days, although there were no statistically significant changes. The average fluorescence intensity of GFAP in the injured area of the siSASH1 group decreased compared with that of the siCtrl group at 7, 14, and 21 days, with a statistically significant reduction at 21 days post‐injury (Figure 2E *p* < 0.05).

The spinal cord tissue mainly consists of neurons and astrocytes; therefore, we wanted to distinguish the effects of SASH1 on neurons versus astrocytes. We firstly investigated the effects of SASH1 depletion on neurons isolated from E14 rat spinal cord, as described in our previous study.^21^ As shown in Figure 2Ga, qRT‐PCR results revealed that SASH1 messenger RNA (mRNA) showed a 45.5% decrease in cultured neurons after SASH1 siRNA treatment for 48 h. Secondly, immunostaining with Tuj1 antibody was performed to investigate the morphological changes in spinal neurons treated with siSASH1 (Figure 2Gb). The results showed no significant alterations in the neuronal survival ratio (Figure 2Gc) or axonal length (Figure 2Gd).

### 3.3 SASH1 siRNA‐treated astrocytes release more BDNF promoting axonal length

**FIGURE 3:**
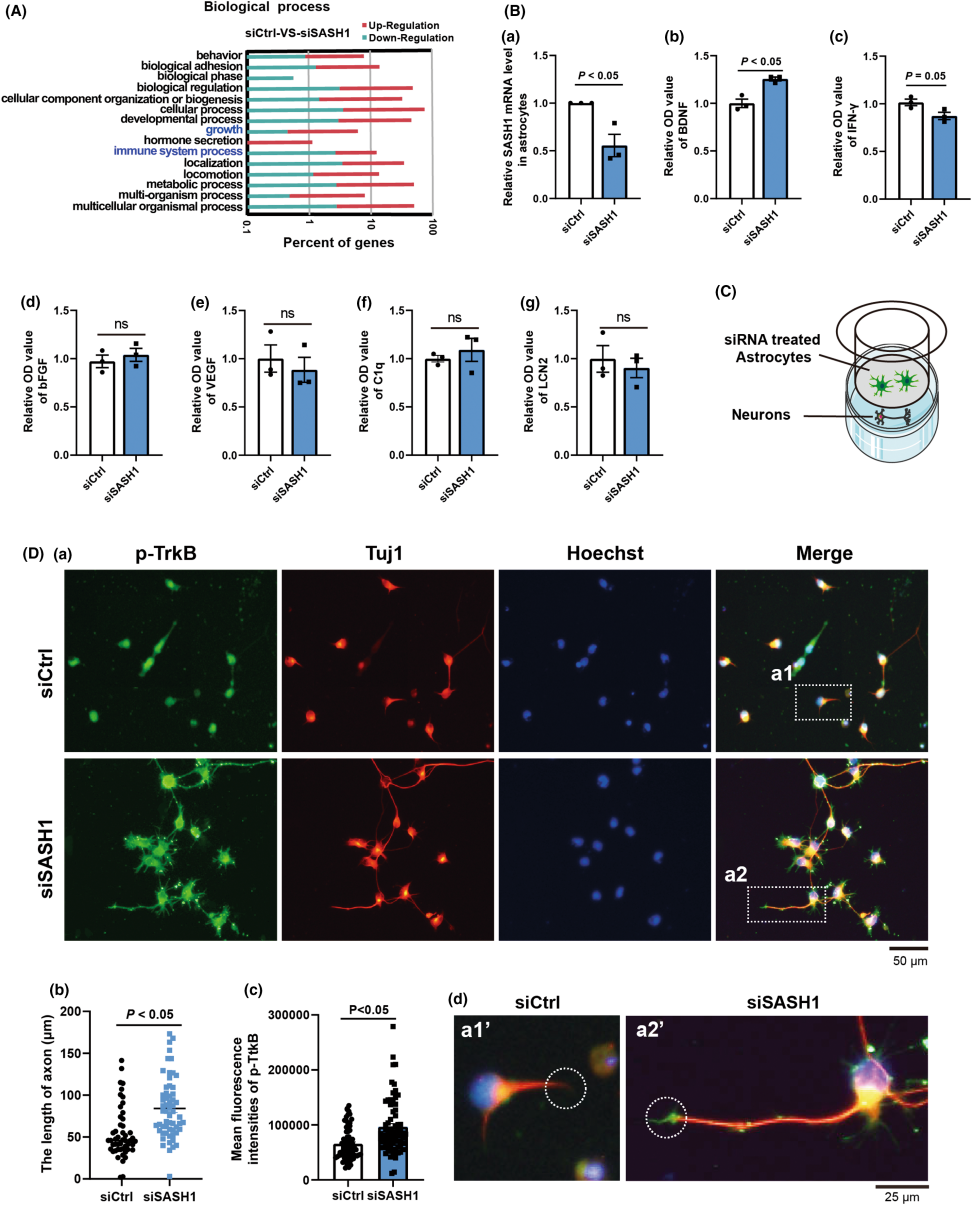
Axonal length increases after coculture with SASH1 siRNA‐treated astrocytes. (A) Fifteen top‐enriched GO terms of the DEGs for biological processes in the cultured astrocytes with siCtrl and siSASH1 treatments. (B Panel a, qRT‐PCR results of SASH1 expression in astrocytes treated with siSASH1. ELISA results for BDNF (panel b), IFN‐γ (panel c), bFGF (panel d), VEGF (panel e), C1q (panel f), and LCN2 (panel g) in the supernatant of cultured astrocytes treated with siCtrl and siSASH1. The *p*‐value is presented in the figure panel, for Student's unpaired *t*‐test of siSASH1 vs. siCtrl. (C) Schematic diagram of the coculture experiment. (D) Immunostaining results of neurons by p‐TrkB and Tuj1 antibodies and Hoechst dye (a). b, Statistical result of the axonal length treated with siCtrl and siSASH1 (axonal length of siCtrl = 54.65 ± 4.32 μm, axonal length of siSASH1 = 84.03 ± 4.71 μm). c, Statistical results of the expression of p‐TrkB treated with siCtrl and siSASH1. d, Expressions of p‐TrkB, panels a1' and a2' are the magnifications of the white squares in panels a a1‐a2, respectively. GO, gene ontology; DEGs, differentially expressed genes; p‐TrkB, phosphorylated‐tropomyosin‐related kinase B; SASH1, SAM and SH3 domain‐containing protein 1; si, small interfering; qRT‐PCR, qualitative real‐time polymerase chain reaction; ELISA, enzyme‐linked immunosorbent assay; BDNF, brain‐derived neurotrophic factor; IFN, interferon; Ctrl, control.

The above results revealed that SASH1 knockdown in neurons did not affect neuronal phenotype, but did affect GFAP expression in vivo. Thus, we investigated the effects of SASH1 depletion in astrocytes isolated from rat spinal cords. In our previous study, we detected alterations in the transcript profiles of rat cortical astrocytes after siSASH1 treatment.^13^ We re‐analyzed the data on biological processes in Gene Ontology (GO) and found that depletion of SASH1 resulted in significant changes in immune system development‐related genes (Figure 3A). Therefore, after using qRT‐PCR to detect the efficiency of SASH1 interference (Figure 3Ba), we measured the expression of some characteristic molecules released form astrocytes known to be either helpful or harmful for axonal growth, including BDNF, bFGF, VEGF, IFN‐γ, LCN2, and C1q, using the ELISA method. As shown in Figure 3Bb, BDNF significantly increased in the culture supernatant, whereas the content of IFN‐γ (Figure 3Bc) decreased in siSASH1‐treated astrocytes. The expression levels of bFGF, VEGF, LCN2, and C1q showed no notable changes (Figure 3Bd–g). These ELISA results implied that SASH1 depletion resulted in the release of certain astrocytic molecules, tending toward the stimulation of neurotrophic astrocytes.

Furthermore, we verified whether siSASH1‐treated astrocytes could improve axonal growth using a transwell chamber. Astrocytes were plated into the upper chamber after siCtrl or siSASH1 treatment for 48 h, while neurons were plated in the lower chamber (Figure 3C). After coculture, the neurons were fixed and immunostained with Tuj1 and TrkB (BDNF receptor) antibodies. As shown in Figure 3Db, the mean axonal length of siCtrl‐treated neurons was 54.65 μm, while that of siSASH1‐treated neurons was 84.03 μm. This represented a 55.6% increase in axonal length compared with that cocultured with siCtrl‐treated astrocytes. The immunostaining results for p‐TrkB revealed a significant increase (Figure 3Dc), especially in the axon tips (Figure 3Dd a1’ and a2’). These above results support that SASH1 plays a role in altering astrocytes function.

### 3.4 SASH1 knockdown increases BDNF during glial differentiation

**FIGURE 4:**
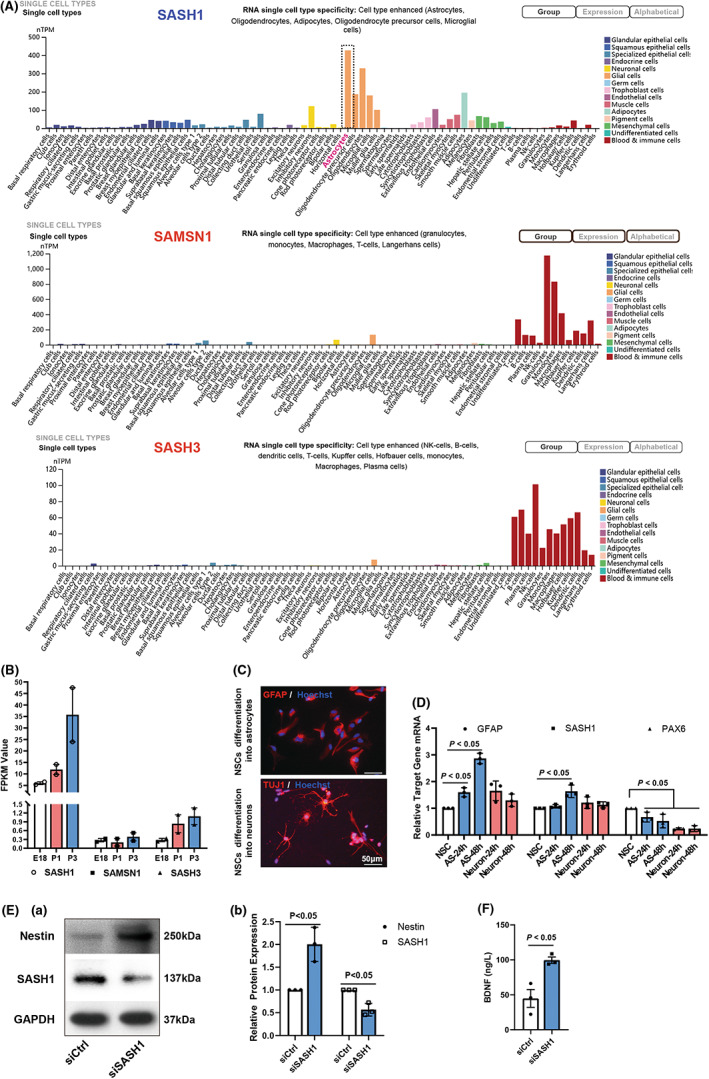
Effect of SASH1 in neural stem cell differentiation. (A) Expression pattern of the SLY family genes in single cell transcriptome. (B) Expression pattern of the SLY family genes during rat brain development. (C) Representative immunofluorescence results of NSCs differentiation into astrocytes or neurons. (D) qRT‐PCR results of GFAP, SASH1 and PAX6 expression during NSC differentiation into neurons and astrocytes for 24 h and 48 h. The target gene mRNA levels in NSCs were normalized as 1, *n* = 3, *p* values presented in figure panel, for Student's unpaired *t*‐test of astrocyte vs. neurons. (E) Western‐blotting results of Nestin and SASH1 of NSCs differentiation into astrocytes with siSASH1 treatment. Panel a, Representative Western‐blotting result. Panel b, Statistical result, *p* values presented in figure panel, for Student's unpaired *t*‐test of SASH1 vs. siCtrl. (F) ELISA results of BDNF in supernatant of NSC differentiation into astrocytes with siSASH1 treatment. *n* = 3, *p* values presented in figure panel, for Student's unpaired *t*‐test of siSASH1 vs. siCtrl. SASH1, SAM and SH3 domain‐containing protein 1; SAMSN1, SAM domain, SH3 domain and nuclear localization signals 1; SASH3, SAM and SH3 domain containing 3; FPKM, fragments per kilo base of transcript per million mapped fragments; E18, embryonic day 18, P1, postnatal day 1; P3, postnatal day 3.

In our previous study,^17^ we detected gene transcript profile alterations in different rat cortical cortex development stages. We became interested in further re‐analyzing the RNA‐seq result and found that SASH1 mRNA increased remarkably during brain development, while its family members, SAM and SH3 domain containing 3 (SASH3) and SAM domain, SH3 domain and nuclear localization signals 1(SAMSN1), did not have this feature (Figure 4B). Thus, we hypothesized that SASH1 may be a pro‐differentiation gene. In addition, with reference to the online database at https://www.proteinatlas.org/humanproteome/single+cell+type, we also found, based on RNA single cell type specificity, that SAMSN1 and SASH3 gene expression groups were enriched in blood and immune cells, while SASH1 has ubiquitous cell types, with its highest expression in astrocytes (Figure 4A). These data suggest that SASH1 plays a unique role in astrocytes and may be a scaffold signaling protein that facilitates astrocyte development.

Therefore, we cultured NSCs in vitro according to our previous study^21^ and then differentiated them into neurons and astrocytes. After differentiation for 24 and 48 h, we detected changes in GFAP, SASH1, and PAX6 gene transcription. The results showed that during differentiation into neurons or astrocytes, PAX6, a transcription factor that maintains stem cell characteristics, was quickly reduced (Figure 4C‐D). GFAP gene expression, as well as SASH1 gene expression, notably increased in astrocytic differentiation, whereas in neuronal differentiation, there were no significant alterations.

Furthermore, we detected changes in the expression of Nestin and SASH1 after siSASH1 treatment during astrocytic differentiation for 48 h. The results shown in Figure 4E reveal that Nestin was maintained at a high level, while the expression of SASH1 was knocked down. We also measured the BDNF content in the culture supernatant of SASH1‐depleted astrocytes and found that the release of BDNF increased remarkably (Figure 4F).

## 4 DISCUSSION AND CONCLUSIONS

CNS injury results in drastic changes in astrocytes, often presenting as a phenotype of astrocytic activation. Astrocytes comprise a majority of cells in the CNS. They are responsible for a wide variety of functions, including the regulation of synaptic activity to prevent damage from spreading.^5^ In the acute and subacute stages of CNS injuries the main functional roles of astrocytes are to maintain the homeostasis of a local microenvironment (neurotransmitters) at the injury site,^22^ regulate neurovascular coupling through the perivascular end feet,^23^, ^24^ affect the blood–brain barrier permeability,^25^ etc. Moreover, astrocytes appear to be important in promoting synaptic plasticity under physiological and/or pathological conditions.^26^, ^27^ Our previous study found that the expression of SASH1 was related to the proliferation, migration, and response of primary cultured astrocytes to the extracellular matrix.^13^ Therefore, in the present study, we investigated the role of SASH1 in SCI.

SASH1 has long been regarded as a tumor suppressor gene, and many publications have reported the loss or downregulation of SASH1 as being related to the promotion of cell proliferation.^28^, ^29^ Our data also support that increased SASH1 expression contributes to glial differentiation. Therefore, we knocked down SASH1 expression in a rat SCI model, and as hypothesized, we found that the depletion of SASH1 decreased astrocytic activation, as evaluated based on GFAP expression levels. Further in vitro spinal neuron and astrocyte culture studies revealed that SASH1 altered astrocyte function but did not directly affect neuronal function. Accordingly, we speculated that SASH1 upregulation is involved in cell differentiation. If this is the case, SASH1 may play a role in NSC differentiation into astrocytes. In fact, the results of NSC differentiation experiments showed an increase in GFAP and SASH1 expression, and the knockdown of SASH1 resulted in increased Nestin protein maintenance, indicating that the downregulation of SASH1 delayed NSC differentiation. Increased BDNF release also matches the characteristics of growth factors that maintain NSC growth.^30^ However, the natural or essential role of SASH1 in astrocytes needs to be investigated in further studies.

The present study revealed that, after SCI, SASH1 protein expression was increased in the spinal cord tissue of rats. When SASH1 was knocked down, rats showed improved recovery of hindlimb motor function, and GFAP expression was significantly reduced. Depletion of SASH1 in cultured spinal neurons did not alter axonal length, and SASH1 knockdown in spinal astrocytes increased BDNF release and decreased IFN‐γ release. Axonal length significantly increased after coculture with siSASH1‐treated astrocytes, while immunostaining for TrkB (BDNF receptor^31^) increased in neurons cocultured with SASH1‐knockdown astrocytes. Further studies found that during differentiation of NSCs into astrocytes, GFAP upregulation along with SASH1 expression increased, but such an effect did not occur in the differentiation of NSCs into neurons. When inhibiting SASH1 expression, differentiating NSCs could maintain Nestin at higher levels and increase BDNF release. Based on our results, we speculated that SASH1 may maintain astrocyte differentiation. Depleting SASH1 delays NSC differentiation and increases BDNF levels. This suggests that SASH1 downregulation may limit glial activation and contribute to the functional recovery from SCI. With the development of neuroscience, combination treatment is recently being explored to address various aspects of SCI pathology and promote discovery of effective treatments.^32^ In future study, we plan to combine alongside SASH1 attenuation to enhance further its efficacy and produce more meaningful outcomes after SCI.

## CONFLICT OF INTEREST

The authors declare no conflicts of interest.

## Supporting information


Data S1

## Data Availability

The data that support the findings of this study are available from the corresponding author upon reasonable request.
