# Supplementary material for: Depletion of SASH1, an astrocyte differentiation‐related gene, contributes to functional recovery in spinal cord injury
Source: CNS Neurosci Ther. 2022 Oct 26;29(1):228–38. doi: 10.1111/cns.13998 (PMC9804067; doi:10.1111/cns.13998)
Supplement: Supplementary file 1 — Data S1 [file CNS-29-228-s001.pdf]

Vazyme (MP102)

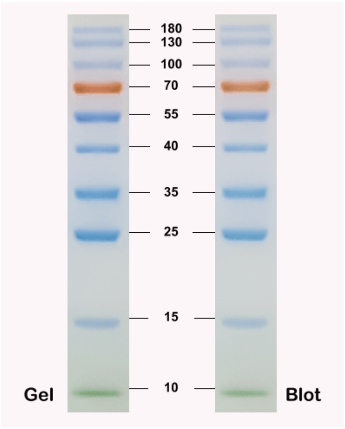

12% Tris-Glycine

Full unedited gel/blot for Figure 1A

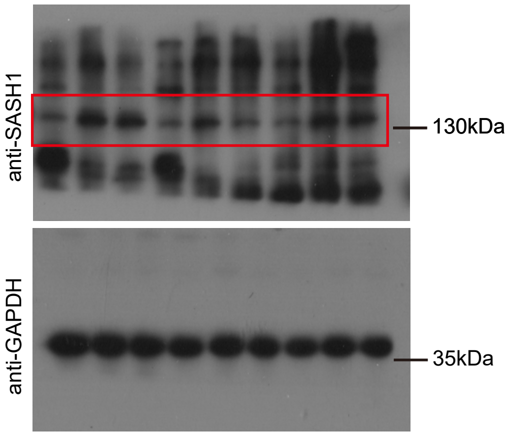

Full unedited gel/blot for Figure 1C

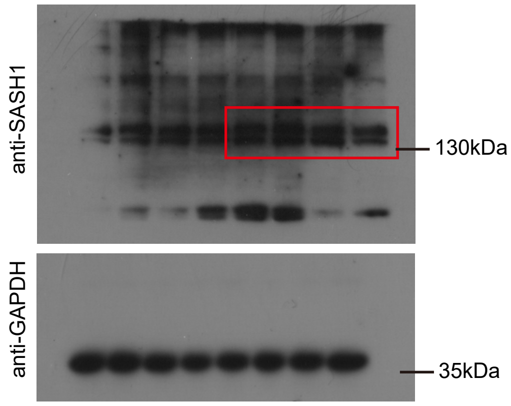

Full unedited gel/blot for Figure 1C

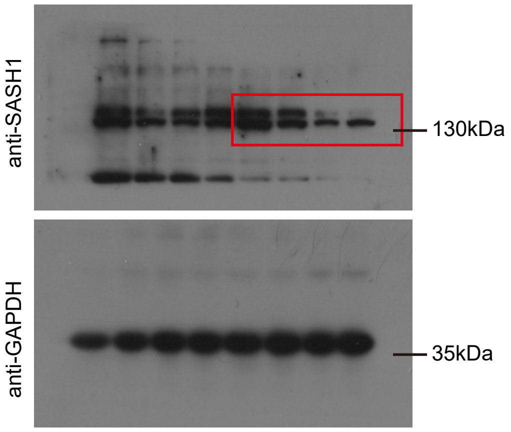

Full unedited gel/blot for Figure 4Ea

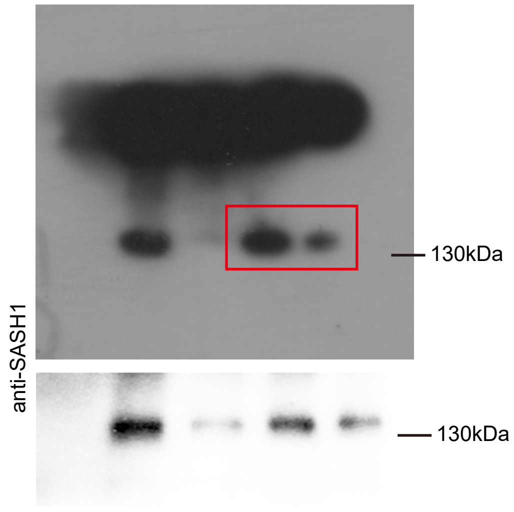

Full unedited gel/blot for Figure 4Ea

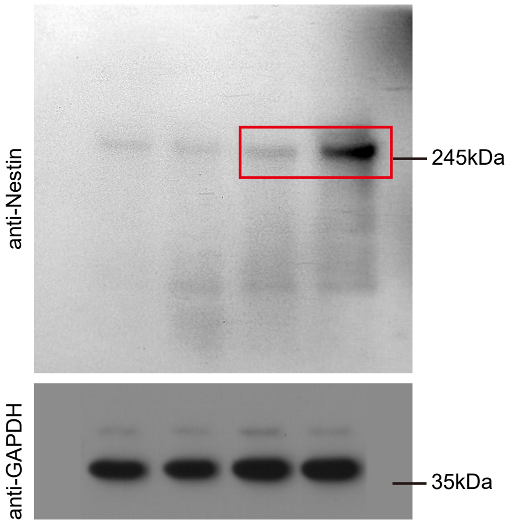

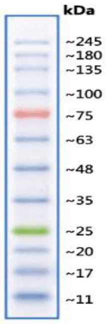

Tris–Glycine
4–20%
